# Exploring the apoptotic effects of sericin on HCT116 cells through comprehensive nanostring transcriptomics and proteomics analysis

**DOI:** 10.1038/s41598-024-52789-8

**Published:** 2024-01-29

**Authors:** Siriluk Ratanabunyong, Jeeraprapa Siriwaseree, Panatda Wanaragthai, Sucheewin Krobthong, Yodying Yingchutrakul, Buabarn Kuaprasert, Kiattawee Choowongkomon, Pornanong Aramwit

**Affiliations:** 1 Department of Pharmacy Practice, Faculty of Pharmaceutical Sciences, Chulalongkorn University, Bangkok, 10330 Thailand; 2 Department of Biochemistry, Faculty of Science, Kasetsart University, Bangkok, 10900 Thailand; 3 Interdisciplinary Graduate Program in Genetic Engineering, Kasetsart University, Bangkok, 10900 Thailand; 4 Thailand Center of Excellence in Natural Products Chemistry (CENP), Department of Chemistry Faculty of Science, Chulalongkorn University, Bangkok, 10330 Thailand; 5 National Center for Genetic Engineering and Biotechnology, NSTDA, Pathum Thani, 12120 Thailand; 6 Synchrotron Light Research Institute (Public Organization), Nakhon Ratchasima, Thailand; 7 Department of Pharmacy Practice, Faculty of Pharmaceutical Sciences and Center of Excellence in Bioactive Resources for Innovative Clinical Applications, Chulalongkorn University, Phayathai Road, Phatumwan, Bangkok, 10330 Thailand; 8 The Academy of Science, The Royal Society of Thailand, Dusit, Bangkok, 10330 Thailand

**Keywords:** Biochemistry, Cancer, Drug discovery, Molecular biology, Health care

## Abstract

Sericin, a silk protein from *Bombyx mori* (silkworms), has many applications, including cosmetics, anti-inflammation, and anti-cancer. Sericin complexes with nanoparticles have shown promise for breast cancer cell lines. Apoptosis, a programmed cell death mechanism, stops cancer cell growth. This study found that Sericin urea extract significantly affected HCT116 cell viability (IC_50_ = 42.00 ± 0.002 µg/mL) and caused apoptosis in over 80% of treated cells. S-FTIR analysis showed significant changes in Sericin-treated cells' macromolecule composition, particularly in the lipid and nucleic acid areas, indicating major cellular modifications. A transcriptomics study found upregulation of the apoptotic signaling genes *FASLG*, *TNFSF10*, *CASP3*, *CASP7*, *CASP8*, and *CASP10*. Early apoptotic proteins also showed that BAD, AKT, CASP9, p53, and CASP8 were significantly upregulated. A proteomics study illuminated Sericin-treated cells' altered protein patterns. Our results show that Sericin activated the extrinsic apoptosis pathway via the caspase cascade (CASP8/10 and CASP3/7) and the death receptor pathway, involving TNFSF10 or FASLG, in HCT116 cells. Upregulation of p53 increases CASP8, which activates CASP3 and causes HCT116 cell death. This multi-omics study illuminates the molecular mechanisms of Sericin-induced apoptosis, sheds light on its potential cancer treatment applications, and helps us understand the complex relationship between silk-derived proteins and cellular processes.

## Introduction

Sericin is a natural polymer found in silk fibers produced by the silkworm *Bombyx mori*. It plays a crucial role in keeping the fibroin filaments together within the silk thread. Sericin can be classified into three fractions, namely sericin A, sericin B, and sericin C, based on their solubility^1^. In silk fibers, Sericin accounts for approximately 20–30% of the total composition, with the remaining portion being fibroin, another protein found in silk^2^. Sericin has found applications in various fields, including medicine and cosmetics, due to its unique physicochemical properties. In the field of biomedicine, Sericin has been studied for its wound-healing properties. It has been shown to promote wound healing and exhibit antimicrobial effects^3–7^. Additionally, Sericin has been utilized in skin, hair, and nail cosmetics. When used in the form of lotions and creams, Sericin has demonstrated increased skin elasticity, anti-wrinkle, and anti-aging effects^8,9^. In a study by Park et al., the effects of silk protein intake on sarcopenia in female Sprague–Dawley rats were investigated. The results indicated that middle-aged female rats who consumed silk protein experienced a decrease in lean body mass and grip strength^10^. Sericin has also shown anti-inflammatory properties by suppressing the production of pro-inflammatory cytokines through pathways involving COX-2 and iNOS^11^. Moreover, Sericin has been explored in cancer therapeutics. It has been incorporated into drug delivery systems for anti-cancer drugs, and studies have demonstrated its potential to enhance the anti-tumor effects of chemotherapy agents^12–14^. Guo et al. developed an amphiphilic polymer containing a Sericin polypeptide backbone, which showed improved efficacy in inducing the anti-tumor effects of Doxorubicin^15^. Furthermore, recent research by Mumtuz et al. highlighted the potential of Sericin complexed with silver nitrate nanoparticles in breast cancer treatment. The study demonstrated that the highest concentration of Sericin complexed with silver nitrate nanoparticles exhibited enhanced anti-proliferative and apoptosis-inducing properties, as well as influencing genetic profiling effects in breast cancer cell lines^16^.

Cancer is characterized by abnormal cell proliferation, which involves the dysregulation of various genes and signaling pathways. Oncogenes, such as Myc, can be upregulated in cancer cells and promote cell proliferation, contributing to tumor growth^17^. On the other hand, tumor suppressor genes like p53 are often downregulated or mutated in cancer, leading to dysfunction in cellular apoptosis signaling^18^. Dysregulation of apoptosis not only affects the development of cancer but also compromises the overall integrity of multicellular organisms. In cases of developmental irregularities, it may lead to autoimmunity, tumorigenesis, and other serious health problems. Tumor cell survival could be induced by activation of anti-apoptotic or inactivation of pro-apoptotic signaling pathways^19,20^. Apoptosis indeed plays a crucial role in inhibiting the progression of cancer cells. It is a vital mechanism for maintaining cellular homeostasis and preventing the uncontrolled growth of abnormal cells^21^. Apoptosis plays a pivotal role in cell homeostasis and separates into two pathways: intrinsic and extrinsic pathways. The intrinsic pathway is mediated via mitochondria to release cytochrome C and promote cell apoptosis. The extrinsic pathway is mediated via the caspase cascade^22^. Once the apoptosis dysfunction occurs, cancer cells can progress. Cancer cells could evade apoptosis by inhibiting caspase function^23^, up-regulating anti-apoptotic BCL-2 proteins^24,25^, and/or loss of BAX or BAK protein to induce apoptosis^26^. All of these promote cancer cell survival and progression. Our preliminary results showed that Sericin extract from urea, heat, acid, and alkali could reduce cancer cell survival (HepG2, HCT116, NCIH-1975, and RBE) (data not shown).

Synchrotron Fourier Transform Infrared (S-FTIR) spectroscopy is a powerful analytical technique used to identify and characterize chemical bonds and functional groups in a wide range of materials^27,28^. It is particularly valuable in the study of organic compounds, including biological samples such as cells, tissues, and biomolecules^29,30^. This study allows for a direct investigation of the molecular changes induced by Sericin in HCT116 cells.

Proteomics and transcriptomics are rapidly growing fields of biochemical study that aim to investigate the complete set of proteins and genes produced by the genome of an organism^31–35^. It involves the identification, quantification, and characterization of proteins and genes and their modifications in complex biological samples. The goal of these omics is to understand the function and regulation of proteins and genes in cellular processes and to link genetic information with functional information. Proteins are central to many biological processes, including metabolic pathways, signal transduction, and regulation of gene expression. Through the examination of proteins, we can acquire an understanding of the fundamental mechanisms of biological activities and detect possible targets for therapeutic intervention.

This study aimed to investigate the mechanism underlying Sericin-induced apoptosis in HCT116 cells, a well-established colorectal cancer cell line. To confirm the effect of Sericin on the survival of HCT116 cells, the Muse analyzer is utilized to determine the level of apoptosis in HCT116 cells treated with Sericin. Additionally, the macromolecule change induced in HCT116 cells upon Sericin treatment could be elucidated through the use of S-FTIR spectroscopy. In addition, to enhance comprehension of the molecular pathways that govern the response of HCT116 cells to Sericin therapy, transcriptomics and proteomics approaches were utilized.

## Results

### IC_50_ determination

**Figure 1:**
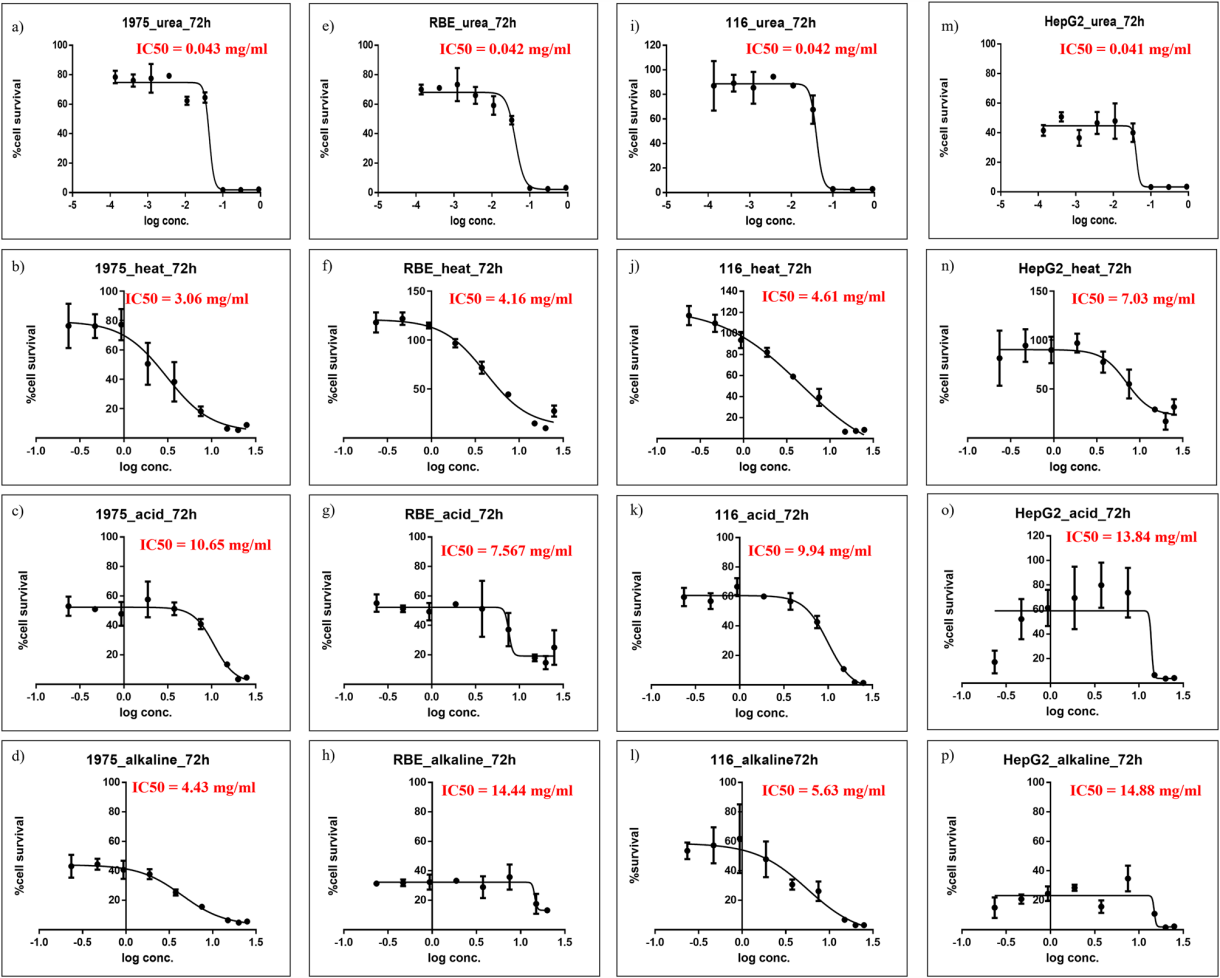
The IC_50_ curve of cancer cells after treatment with Sericin for 72 h. (**a**–**d**) represent the NCI-H1975 cells against Sericin that were extracted by urea, heat, acid, and alkaline, respectively. (**e**–**h**) were presented for the RBE cell against Sericin that was extracted by urea, heat, acid, and alkaline, respectively. (**i**–**l**) were HCT116 cell-treated Sericins that were extracted by urea, heat, acid, and alkaline, respectively. (**m**–**p**) represent the HepG2 cells against Sericin that are extracted by urea, heat, acid, and alkaline, respectively.

The experiment aimed to determine the effect of Sericin extract on cancer cells using a cell viability assay. Four types of cancer cells, namely NCI-H1975, RBE, HCT116, and HepG2 cells, were selected for the study. The results demonstrated that all Sericin samples exhibited cytotoxicity on the cancer cells, with IC_50_ values ranging from 40 to 15,000 µg/mL. Among the tested samples, the Sericin extract prepared with urea showed the highest activity against all four types of cancer cells. They were 43, 42, 42, and 41 µg/mL for NCI-H1975, RBE, HCT116, and HepG2 cells, in that order, as shown in Table 1 and Fig. 1. The results suggested that Sericin extract, particularly after being prepared with urea, possesses significant cytotoxic activity against various cancer cell lines, including NCI-H1975, RBE, HCT116, and HepG2 cells. Indeed, the selection of HCT116 cells for further experimentation is justified due to their high sensitivity to the Sericin-urea extract treatment observed in the cell viability assay. These findings indicate that HCT116 cells are responsive to the toxic effects of Sericin-urea, making them a suitable model for detailed investigation. Further investigations are necessary to understand the processes that cause the cytotoxic effects of Sericin extract and to develop ways for detecting apoptosis.

**Table 1:** The IC_50_ values for Sericin samples on cancer cells were determined 72 h post-treatment.

Cancer cells	Sericin IC_50_ (µg/ml)			
	Urea	Heat	Acid	Alkaline
NCI-H1975	43 ± 0.001	3060 ± 0.53	10,650 ± 0.096	4430 ± 0.47
RBE	42 ± 0.001	4160 ± 0.45	7570 ± 0.99	14,440 ± 2.79
HCT116	42 ± 0.002	4610 ± 0.61	9940 ± 0.07	5630 ± 1.09
HepG2	41 ± 0.004	7030 ± 1.46	13,840 ± 0.77	14,880 ± 0.12

### Apoptosis detection

**Figure 2:**
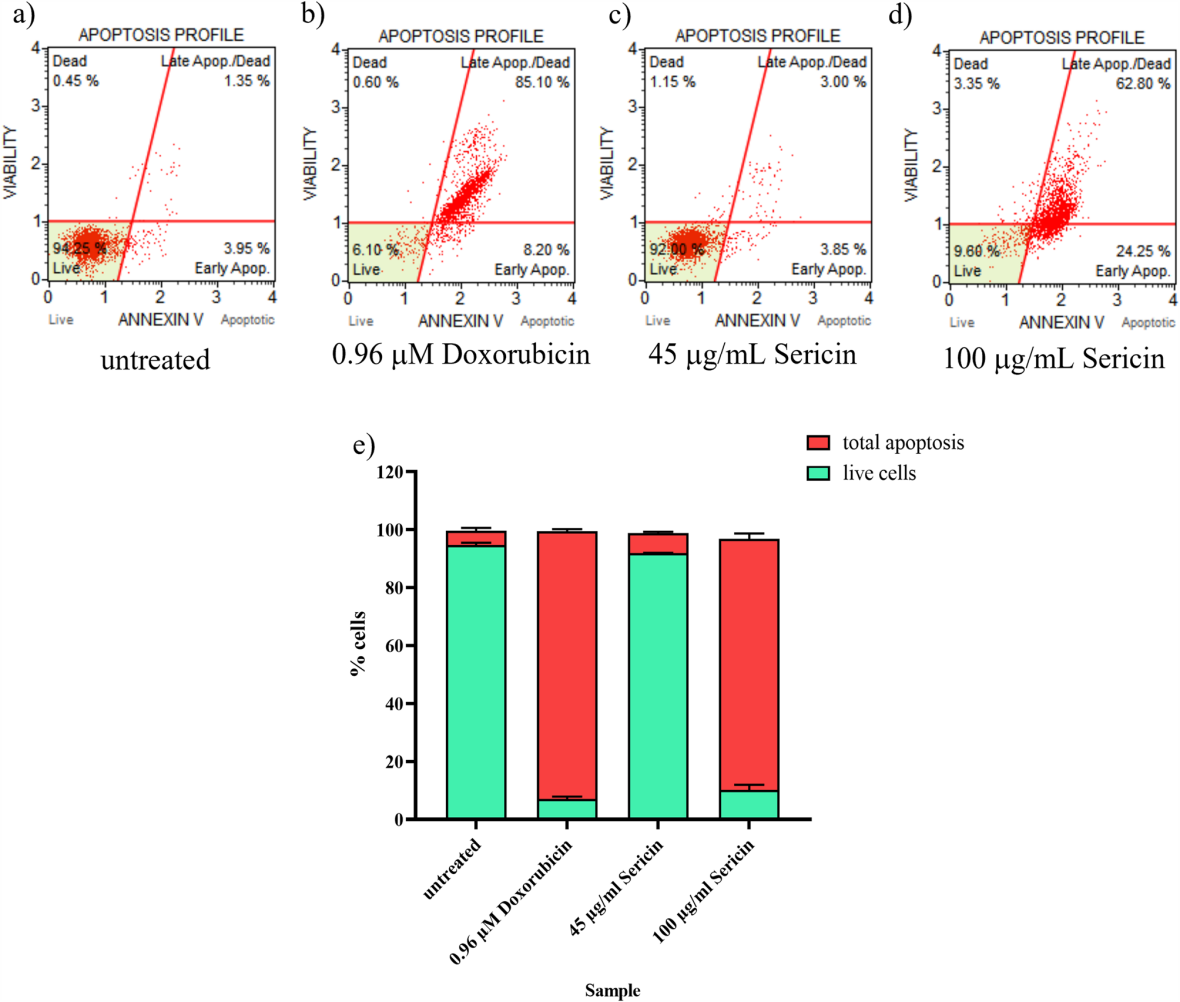
The apoptosis profile of HCT116-treated cells. (**a**) untreated; (**b**) treated with Doxorubicin at 0.96 µM; (**c**, **d**) treated with Sericin at 45 and 100 µg/mL, respectively. (**e**) Summary of % apoptosis on HCT116 apoptotic cells after treatment for 72 h.

Apoptosis cell death detection by the Muse® Annexin V & Dead Cell Kit was used to detect apoptotic cell death in HCT116 cells treated with Sericin. The experiment aimed to measure the effect of Sericin on apoptosis induction in HCT116 cells, comparing it with the known apoptotic inducer Doxorubicin (Fig. 2). The HCT116 cells were treated with different concentrations of Sericin and Doxorubicin based on their respective IC_50_ values. After a 72 h treatment period, the HCT116 cells were harvested and stained using the apoptosis detection kit. The results indicated that Sericin treatment at a concentration of 100 µg/mL, the highest concentration, increased early apoptosis in HCT116 cells from 3.40% (untreated cells) to 23.70% and late apoptosis to 62.97% (Fig. 2d). Doxorubicin, used as the apoptosis control, induced early apoptosis to 8.23% and late apoptosis to 84.13% (Fig. 2b). These findings demonstrated that Sericin affected HCT116 cancer cell apoptosis in a dose-dependent manner. The results implied that higher concentrations of Sericin led to an increased induction of both early and late apoptosis in HCT116 cells. Further studies would be required to investigate the underlying mechanisms of apoptosis induction by Sericin.

### Determination of macromolecule change by Synchrotron Fourier transform infrared (S-FTIR) spectroscopy

**Figure 3:**
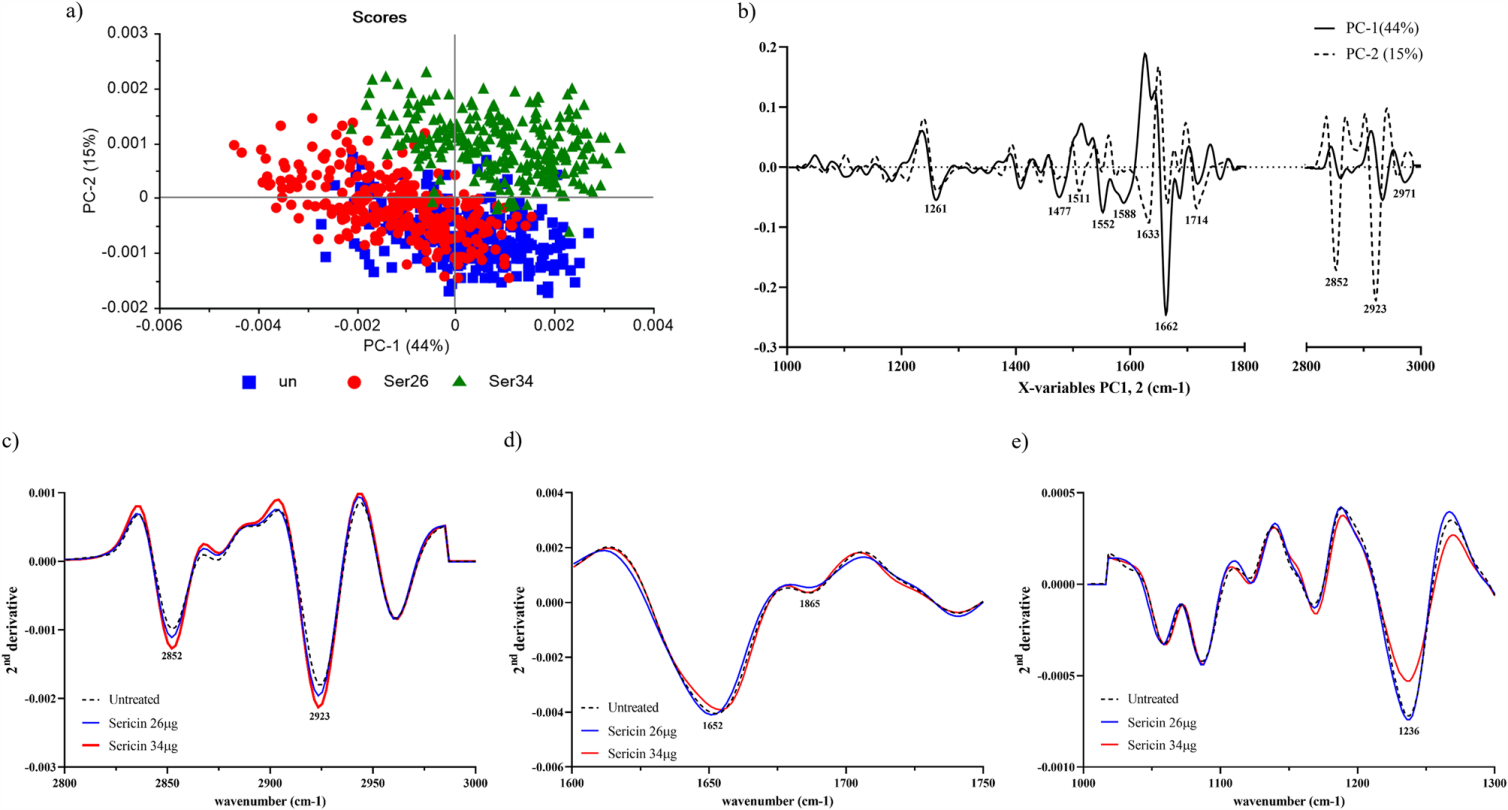
Principal Component Analysis (PCA) was conducted on S-FTIR data. HCT116 cells were treated at different concentrations (26 and 34 µg/mL) within the spectral range of 3000–2800 cm^−1^ and 1800–900 cm^−1^ regions. In the PCA analysis, PC1 and PC2 accounted for 44% and 15% of the total variance, respectively. (**a**) The PCA 2D score plot illustrated the distribution of samples based on PC1 and PC2. (**b**) The loading plot displayed the contributions of PC1 and PC2 to the overall spectral variations. (**c**–**e**) The second derivative spectra of the lipid regions (3000–2800 cm^−1^), amide regions (1740–1600 cm^−1^), and nucleic acid, and other carbohydrate regions (1300–1000 cm^−1^) were averaged separately.

In a study, the macromolecule changes in HCT116 cells were measured using S-FTIR spectroscopy. Based on the PCA plot (Fig. 3a), the group of HCT116 cells that were treated with 34 µg/mL of Sericin could be separated from the group of cells that were not treated because their macromolecules had changed. These findings indicate that the use of Sericin therapy resulted in noticeable changes in the cells' macromolecules, as identified by the S-FTIR approach. Nevertheless, the alterations in macromolecules caused by the administration of Doxorubicin to the HCT116 cells were not observable using the S-FTIR approach (unavailable data). The absence of spectral signals in the S-FTIR analysis for Doxorubicin-treated cells may be attributed to substantial damage or cell death, potentially through processes such as apoptosis, rendering the spectral signals undetectable. The loading plot showed the changes in lipid, amide, and nucleic acid (Fig. 3b). The spectral differences were observed mainly in lipid regions centered at 2923 cm^−1^ and 2852 cm^−1^ (Fig. 3c), amide regions centered at 1652 cm^−1^ and 1865 cm^−1^ (Fig. 3d), and nucleic acid and other carbohydrate regions centered at 1236 cm^−1^ (Fig. 3e). The level of lipid content was increased in the Sericin-treated group at a concentration of 34 µg/mL compared to the untreated group. Changes in lipid accumulation and cell membrane in HCT116 cells might be the cause of the increase in lipid content. The cellular protein change was found to be a little bit higher in Sericin-treated cells. There was a difference in the S-FTIR spectra of the untreated and Sericin-treated groups in the nucleic acid regions, with less DNA content in the treated group. The reduction in DNA content may be attributed to cellular apoptosis. Next, a transcriptomics analysis was done to better understand the apoptosis pathway that is involved in cells that were treated with Sericin-HCT116.

### NanoString-based transcriptomics data processing and pathway analysis

Transcriptomics analysis is a valuable tool for studying gene expression patterns and identifying differentially expressed genes in response to specific treatments or conditions. In the context of investigating the apoptotic pathway involved in Sericin-HCT116-treated cells, transcriptomics analysis can provide insights into the gene expression changes that occur during apoptosis. RNA samples were analyzed using the NanoString panel, measuring the expression levels of 770 genes from human canonical pathways. These genes covered various biological pathways such as DNA damage repair, cell cycle, and apoptosis. To achieve a more comprehensive view of the reproducibility of RNA measurements, QC was assessed using pairwise correlation analysis between two experimental groups (Fig. S1). RNA correlation analysis determines the regression analysis between the Sericin versus untreated and Doxorubicin versus untreated groups using the correlation coefficient (R-square, R^2^). The R^2^ value provides a means of evaluating the strength of the relationship between the RNA count data from one group and the RNA count data from the other group. The RNA correlation analysis between Sericin versus untreated groups and Doxorubicin versus untreated groups revealed an R^2^ of 0.958 and 0.961, respectively. These values would indicate a strong linear relationship between the two groups. This result could suggest that the sample preparation was done correctly, leading to consistent and reliable RNA count data. The results of the RNA count analysis revealed a diverse expression profile of genes across the samples. We found 339 genes were differentially up-regulated, whereas 407 genes were down-regulated in the Doxorubicin-treated compared to the untreated experiment. For a comparison of Sericin compared to an untreated experiment, we found 320 genes were differentially up-regulated, whereas 426 were down-regulated. The differential expression of these genes was mapped using PADOG analysis, as shown in Fig. S2.

**Figure 4:**
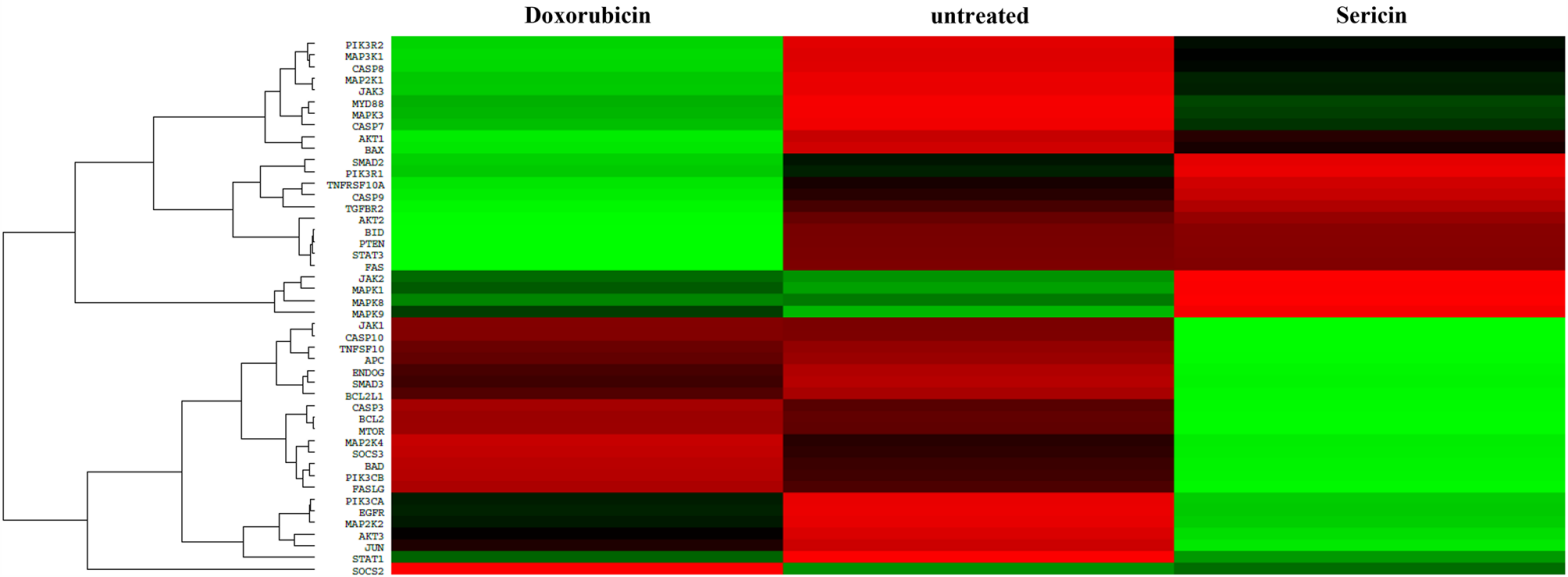
Heat map analysis of HCT116-Sericin-treated RNA. HCT116 cells were treated with 45 µg/mL Sericin for 24 h. Following that, total RNA was extracted and gene expression was determined. The green color represents an upregulated gene. The red color represents a downregulated gene. The black color showed the unchanged gene.

The analysis of gene expression data using nanostring Gene Expression CodeSet RNA hybridization and heat map analysis revealed interesting findings in HCT116-Sericin-treated cells compared to untreated and Doxorubicin-treated cells (Fig. 4 and Table S1). The findings from the gene expression analysis provide valuable insights into the effects of Sericin treatment on HCT116 cells. The upregulation of genes involved in the apoptotic pathway, such as *FASLG*, *TNFSF10*, *CASP3*, *CASP7*, *CASP8*, and *CASP10*, suggests that Sericin treatment induces apoptosis in these cells. The binding of death ligands to death receptors on the cell surface triggers the extrinsic apoptosis pathway, and the upregulation of *CASP3*, *CASP7*, *CASP8*, and *CASP10* indicates this. In contrast, the upregulation of the anti-apoptotic proteins BCL2 and BCL2L1 was also detected. These proteins are known to be involved in inhibiting apoptosis and promoting cancer cell survival. In colorectal cancer and other types of cancer, the intrinsic apoptosis pathway is often out of whack, which means that anti-apoptotic proteins like BCL2 and BCL2L1 are overexpressed. This dysregulation contributes to the evasion of apoptosis and promotes cancer cell progression^36^. The results suggested that many molecules interplay between different factors and signaling pathways in the response of HCT116-Sericin-treated cells. To further investigate the mechanism of apoptosis induction by Sericin in HCT116 cells, we proposed to determine the levels of early apoptotic proteins using the MILLIPLEX® Early Apoptosis 7-Plex Magnetic Bead assay.

### Early apoptotic protein detection by MILLIPLEX® early apoptosis 7-Plex

**Figure 5:**
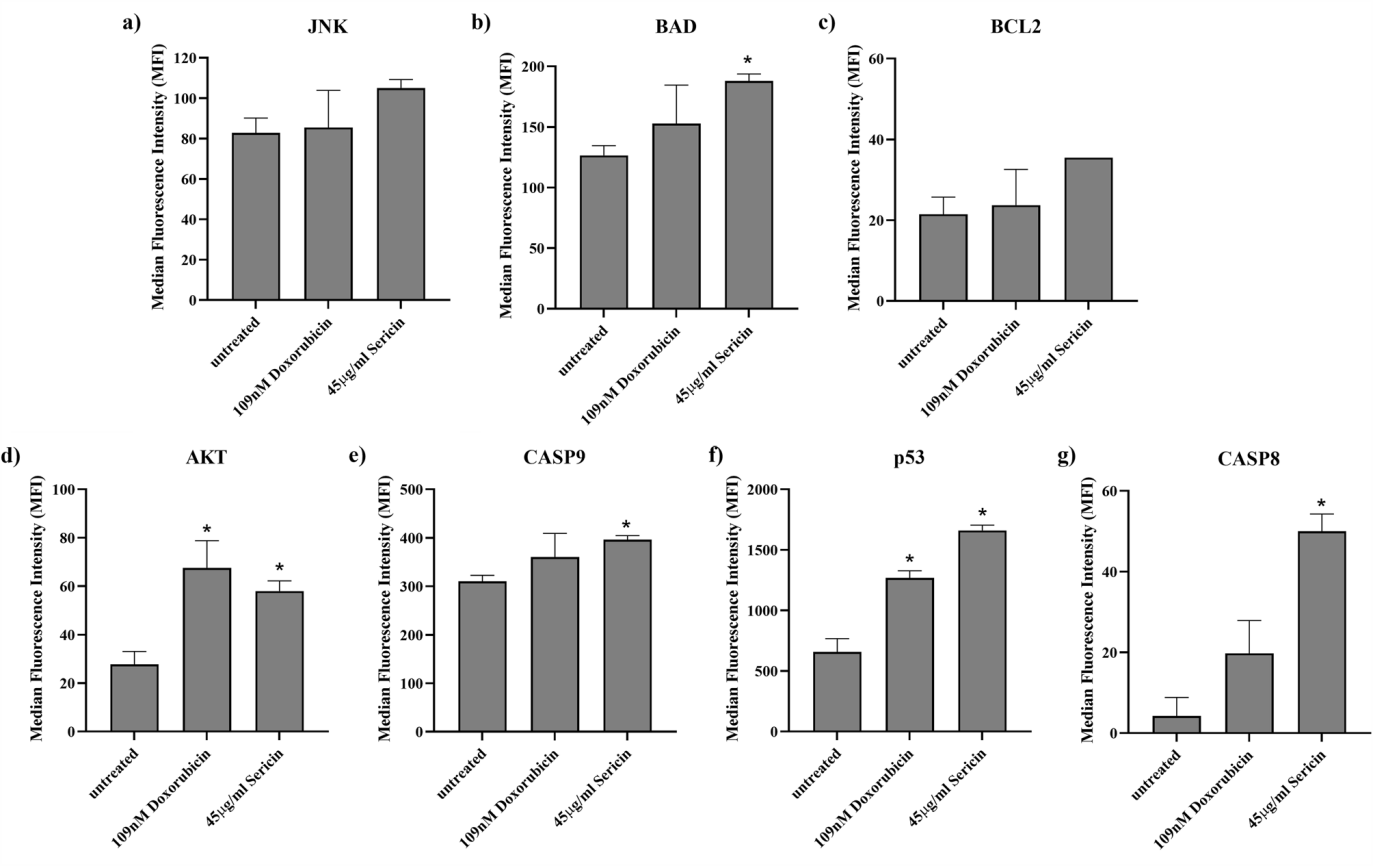
The level of early apoptosis protein in HCT116 cells. (**a**) JNK, (**b**) BAD, (**c**) BCL2, (**d**) AKT, (**e**) CASP9, (**f**) p53, and (**g**) CASP8 protein expression in HCT116-treated cells. The y-axis represents the Median Fluorescence Intensity (MFI), and the x-axis represents the sample list. All experiments were carried out in at least three independent replicates (n = 3), and a multiple t-test was used (*p*-values < 0.05).

The MILLIPLEX® Early Apoptosis 7-Plex assay was conducted to verify the impact of Sericin on apoptosis in HCT116 cells. We used this test to see how much of seven early apoptotic proteins (JNK, BAD, BCL2, AKT, CASP9, p53, and CASP8) were present in sericin-treated HCT116 cells. According to the results obtained from the MILLIPLEX® assay, Sericin treatment induced overexpression of several early apoptotic proteins compared to untreated cells. Specifically, BAD, AKT, CASP9, p53, and CASP8 were found to be significantly upregulated in response to Sericin treatment (Fig. 5). This finding was consistent with the transcriptomics results and provided direct evidence supporting the role of Sericin in inducing apoptosis in HCT116 cells. The upregulation of BAD, AKT, CASP9, p53, and CASP8 proteins indicated that Sericin treatment could promote early apoptotic processes in HCT116-treated cells. These proteins play important roles in the apoptotic signaling pathway. BAD is a pro-apoptotic protein that promotes cell death, while AKT is an anti-apoptotic protein that inhibits apoptosis^37,38^. The upregulation of CASP9, p53, and CASP8 further suggests the activation of apoptotic pathways, as these proteins are involved in caspase-dependent cell death and regulation of cell cycle checkpoints^39–41^. In the next experiment, proteomics profiling was conducted to obtain the protein profile in HCT116 treated cells.

### Proteome profiling of HCT116-Sericin treated cells

**Figure 6:**
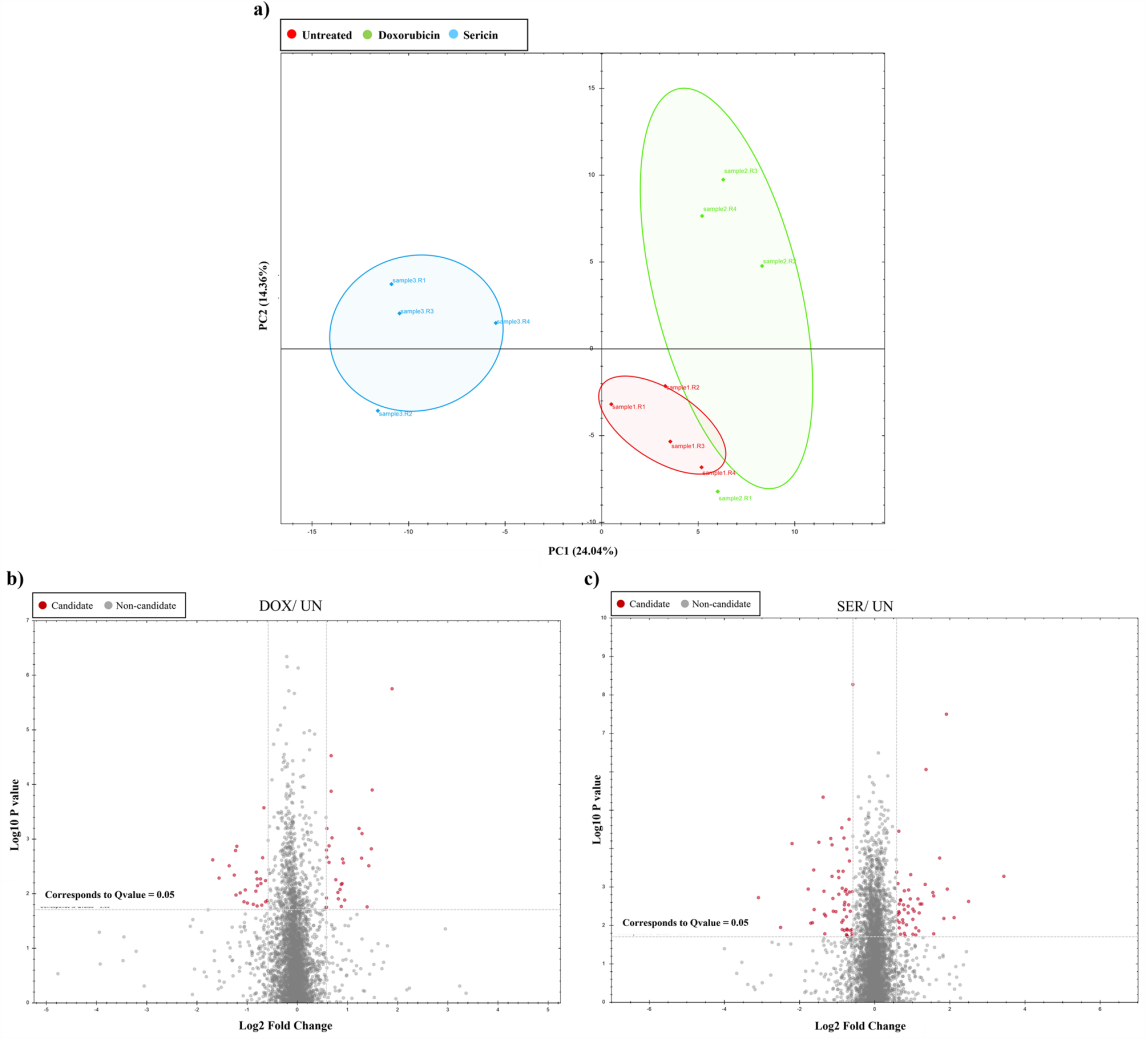
PCA analysis and Volcano plot (**a**) PCA shows the clustering of the samples based on their protein profiles. (**b**, **c**) The plot shows the results of the differential abundance test by plotting the proteins' fold changes against the significance level. The red color indicates the up-and-down regulation and percentage of change in expression. (**b**) Doxorubicin versus untreated; (**c**) Sericin versus untreated. *DOX* Doxorubicin, *SER* Sericin, *UN* untreated.

To illustrate the trend of overall proteome profiling, PCA was conducted. PCA analysis finds clusters or groups of samples based on the amounts of proteins they contain. This helps researchers figure out how different experimental conditions are related and how they are similar. In this case, the PCA clustering analysis highlights the distinct protein abundance profile of the Sericin-treated group compared to the untreated and Doxorubicin groups. The results of the PCA clustering analysis, as shown in Fig. 6a, indicate that the untreated group and Doxorubicin-treated cells are closely clustered together, suggesting that they share similar protein abundance profiles. On the other hand, the Sericin-treated group appears to be farther apart from the other groups, indicating a distinct protein abundance pattern compared to the untreated and Doxorubicin groups. These results indicate that the experimental conditions, particularly the administered treatments (Sericin and Doxorubicin), exerted a substantial influence on the abundance levels of proteins. The discernible variations in protein profiles among the untreated, Doxorubicin, and Sericin groups are not likely solely due to technical variability but rather signify authentic distinctions induced by the respective treatments. In summary, these findings suggest that Sericin treatment brings about distinct alterations in the proteome of HCT116 cells.

The LC–MS/MS analysis found 3131 proteins (2923 protein groups) in the Sericin treatment group and the control group (Doxorubicin and untreated). To further analyze the differential protein expression, the proteome dataset was normalized. The normalized proteomic analysis generated a volcano plot, as shown in Fig. 6b and c. The volcano plot is a commonly used visualization tool to identify proteins with significant changes in expression between two groups. Each spot on the volcano plot represents the log2 protein expression ratio between the Sericin treatment and control groups, plotted against the log10 of the *p*-values associated with the protein expression changes. The spots on the volcano plot indicate the differential expression of proteins, with those located further away from the center and higher up or down indicating greater significance and fold change. Specifically, proteins with a log2 fold change greater than 1 (or less than − 1) are considered differentially expressed. The QC of the proteomics dataset is shown in Fig. S3.

Distinct expression profiles were observed when comparing Doxorubicin-treated samples with the untreated group and Sericin-treated samples with the untreated group. In the Sericin treatment group, a total of 105 proteins exhibited significant differential expression. Notably, Endoplasmic reticulum-Golgi intermediate compartment protein 2 demonstrated the highest upregulation (*p*-value = 0.0005), while 60S ribosomal protein L29 exhibited the lowest downregulation (*p*-value = 0.004), as shown in Table S2. In particular, ELOVL5 (elongation of very long chain fatty acid protein 5) was upregulated. Based on the information provided, it seems that the S-FTIR results have shown an increase in lipid content in Sericin-treated cells. The possible cause of this increment is suggested to be the effect of ELOVL5. On the other hand, the Doxorubicin treatment group showed differential expression in 53 proteins. Remarkably, apolipoprotein E displayed the highest upregulation (p-value < 0.0001), whereas AP-2 complex subunit sigma exhibited the lowest downregulation (*p*-value = 0.002).

## Discussion

This research aimed to elucidate the mechanisms driving apoptosis induced by Sericin in HCT116 cells, a widely recognized colorectal cancer cell line. The study employed omics tools, specifically transcriptomics and proteomics analyses, to comprehensively investigate and understand the molecular processes involved in Sericin-induced apoptosis in these cells. The extraction methods for sericin, a protein derived from silk, vary based on its properties and intended applications. Heat treatment involves boiling silk fibers in water, though it may not yield high purity. Alkali extraction uses an alkaline solution on silk fibers, risking protein degradation under strong alkaline conditions. Acid extraction, using acidic conditions, requires careful control to avoid protein degradation. A urea solution is used to dissolve Sericin. Aramwit found that Sericin-urea extracts were more toxic to cell viability than other methods^42^. Cherdchom reported that major thermal degradation of Sericin occurred between 200 and 400 °C due to heat-breaking peptide bonds and amino acid residues. However, urea-extracted Sericin exhibited less thermal stability and variable degradation profiles compared to other methods, suggesting potential changes in composition and structural characteristics^43^. The observed differences in thermal behavior and stability among extraction methods imply that the extraction process influences Sericin's properties. The specific composition, structural modifications, and thermal stability obtained through different extraction methods have implications for Sericin's applications in material science or biomedicine. The choice of extraction method depends on the intended application, such as in cosmetics, textiles, or biomedical fields. This research selected Sericin-urea extract to illustrate its impact on apoptosis in HCT116 cells. There was some therapeutic evidence for treating cancer with Sericin. Various studies found the apoptotic effects of Sericin on human cancer cells, such as that utilizing human colon cancer cells (SW480) with Sericin led to induction of apoptosis, decreased cell viability, caspase-3 activity, and decreased Bcl-2 expression^44^. Silk Sericin increased intracellular ROS levels in cells A431, SAS, and MCF-7, causing cell cycle arrest during the sub-G1 phase and induced apoptosis^45^. These findings indicate that Sericin holds the potential to instigate apoptosis in diverse cancer cell types, suggesting its prospective therapeutic applications in cancer treatment. However, a more in-depth study using transcriptomics, proteomics, and changes in macromolecules is needed to fully understand how these effects work and to find out if Sericin is safe and effective in treating cancer.

To identify apoptosis, we employed the concentration of Doxorubicin acquired from Khaleel Sahar A^46^. The IC_50_ value of Doxorubicin on HCT116 cells was found to be 0.96 µM. This study employed identical concentrations and assessed the effects 72 h post-treatment, potentially indicating an extended presence of Doxorubicin under certain conditions. The previous study showed that the apoptotic response of HCT116 cells is dependent on time^47^. For the Sericin treatment, we utilized a concentration equal to the IC_50_ value. At this concentration, there was a minimal impact on apoptosis. However, when the dosage was increased to 100 µg/mL, the detection of apoptosis increased by over 80%. This indicates that Sericin exhibited a dose-dependent effect on inducing apoptosis in HCT116 colon cancer cells. In addition, molecules involved in the apoptosis of cells treated with HCT116-Sericin were looked at in this study. The results from transcriptomics and early apoptotic proteins align with previous findings, suggesting that Sericin influences apoptosis in HCT116 colon cancer cells through the death receptor pathway, thereby activating the extrinsic apoptotic pathway.

S-FTIR is a widely utilized analytical technique that can provide valuable insights into lipid, protein, and nucleic acid profile changes induced by exogenous compounds^48^. This technique involves the examination of infrared spectra generated by the vibrational transitions of molecular bonds within the protein structure. Through the analysis, S-FTIR spectra can gain information on the secondary and tertiary structures of proteins and identify changes in protein conformation due to interactions with bioactive compounds. The finding of increased lipid content in the Sericin-treated group compared to the untreated group may involve the upregulation of the elongation of very long chain fatty acids protein 5 (ELOVL5) observed in the proteomics results. ELOVL5 is known to be involved in the biosynthesis of very long-chain fatty acids (VLCFAs) through the process of fatty acid elongation^49^. VLCFA functions include skin barrier formation, liver homeostasis, myelin maintenance, spermatogenesis, retinal function, and anti-inflammation through their influences as components of membrane lipids, sphingolipids, and glycerophospholipids, or precursors of inflammation-resolving lipid mediators. VLCFAs are synthesized by endoplasmic reticulum membrane-embedded enzymes using ELOVL1-7 as a substrate^50^. Also, del Solar et al. used untargeted comparative lipidomics to look into how the composition of lipids changed when staurosporine caused apoptosis in HCT-116 cells. They demonstrated that ceramides, dihydroceramides, and sphingomyelins are involved in staurosporine-HCT116 cell apoptosis^51^. The changes in lipid accumulation and cell membrane composition in response to Sericin treatment may have important implications for cellular functions and signaling pathways. Based on the S-FTIR spectra for DNA at wavenumber 1236 cm^−1^ this represents an O–H or N–H deformation^52^. The O–H or N–H deformation can be influenced by various factors, including hydrogen bonding, solvent environment, and interactions with other molecules or ions. Analytical techniques such as S-FTIR spectroscopy can be employed to detect and analyze these vibrational modes, offering insights into the structural changes occurring during DNA degradation. DNA degradation constitutes a prominent feature of cell apoptosis. The fragmentation and breakdown of DNA during apoptosis contribute to the controlled removal of unwanted or damaged cells. Endonucleases, including enzymes like caspases, cleave DNA at specific sites, orchestrating DNA degradation in apoptosis. These endonucleases target genomic DNA, leading to its fragmentation into smaller fragments. From the provided information, it can be inferred that a reduction in DNA content was observed in HCT116 cells treated with Sericin.

Our finding revealed that the gene expression pattern of Sericin-treated cells was different from that of untreated cells. The results showed that Sericin could induce many gene expressions, including *FASLG*, *TNFSF10*, *CASP3*, *CASP7*, *CASP8*, and *CASP10*, which are involved in the extrinsic apoptotic pathway. HCT116 cells, the colon cancer cells, resisted apoptosis, in which many genes involved in the apoptotic pathway were upregulated and downregulated, as shown in Fig. 4. HCT116-Sericin-treated cells at the IC_50_ concentration (45 µg/mL) induced lower apoptosis, maybe due to the expression of the anti-apoptotic gene in HCT116, whereas increasing the concentration of Sericin (100 µg/mL) could induce HCT116 cell apoptosis (Fig. 2). The interplay between anti-apoptotic and pro-apoptotic factors, as well as the activation of extrinsic apoptosis pathways, may contribute to the observed resistance to apoptosis and the potential for increased apoptosis at higher concentrations of Sericin. In a study by Wang et al., they studied the effect of Bufalin on HCT116 apoptosis cells. They concluded that Bufalin induced apoptosis via the activation of *PTEN*, *AKT*, *Bad/Bax*, and *CASP3* genes in intrinsic apoptotic pathways^53^. In another study by Kim et al., they found that Artemisia annua Linné extract could induce apoptosis by causing a mitochondrial event. These activations occur via the regulation of proteins such as Bax, Bak, and cytochrome c in PDK1/Akt signaling pathways through a PTEN/p53-independent manner^54^. Chen et al. studied tetrandrine as an anticancer property in HCT116 cells. They concluded that tetrandrine may be partly mediated by TGF-β1 by upregulating its expression. It may inactivate the PI3K/Akt signaling by partly reducing PTEN phosphorylation and inhibiting cancer cell proliferation^55^. Zhang et al. proved the property of UNBS5162, which promotes HCT116 cell apoptosis by downregulating Bcl-2 expression and activating Bax and active CASP3 overexpression^56^. Additionally, Chiu et al. investigated the effect of 6,7-dihydroxy-2-(4′-hydroxyphenyl) naphthalene (PNAP-6) on HCT116 cells. They concluded that PNAP-6 caused cells to stop in the G2/M phase and increased caspase activation through pathways for extrinsic apoptosis and ER stress. PNAP-6-induced Fas protein and ER stress marker expression^57^. In contrast to the cells treated with Doxorubicin, the outcomes revealed a distinct pattern of gene expression between Doxorubicin-treated cells and those treated with Sericin. The mechanism of action for Doxorubicin involves the induction of p53, which subsequently activates the upregulation of *BAX*. Increased *BAX* levels lead to the release of cytochrome c from mitochondria, activating caspases, particularly *CASP9*, thereby initiating the intrinsic apoptotic pathway. *CASP7*, identified as a downstream effector caspase, is then activated, culminating in the execution of apoptosis. This finding aligns with the report by Zhou, Y. Y.^58^.

Drawing from various studies and our transcriptomics findings, it is plausible to infer that HCT116 cells treated with Sericin can induce programmed cell death in colon cancer cells, and the efficacy of this effect is contingent on the treatment dosage. These triggers may involve the death receptor pathway, specifically through *TNFSF10* or *FASLG*, and activate *CASP8*, *CASP10*, *CASP3*, and *CASP7* in the extrinsic apoptosis pathway. To substantiate the apoptosis-inducing capability of Sericin on the cells, the levels of early apoptotic proteins were assessed. The results confirmed that Sericin can initiate the early production of proteins associated with apoptosis, including CASP9, p53, and CASP8, aligning with the transcriptomics results. In summary, Sericin impacts apoptosis in HCT116 cells through the extrinsic apoptosis pathway in a dose-dependent manner via the death receptor pathway. The results, which encompassed cytotoxicity, S-FTIR, transcriptomics analysis, early apoptotic protein detection using MILLIPLEX® Early Apoptosis 7-Plex detection, and proteomics analysis, clearly showed that Sericin-urea triggered apoptosis in HCT116 cells through the extrinsic apoptosis pathway, via death receptors.

## Conclusion

**Figure 7:**
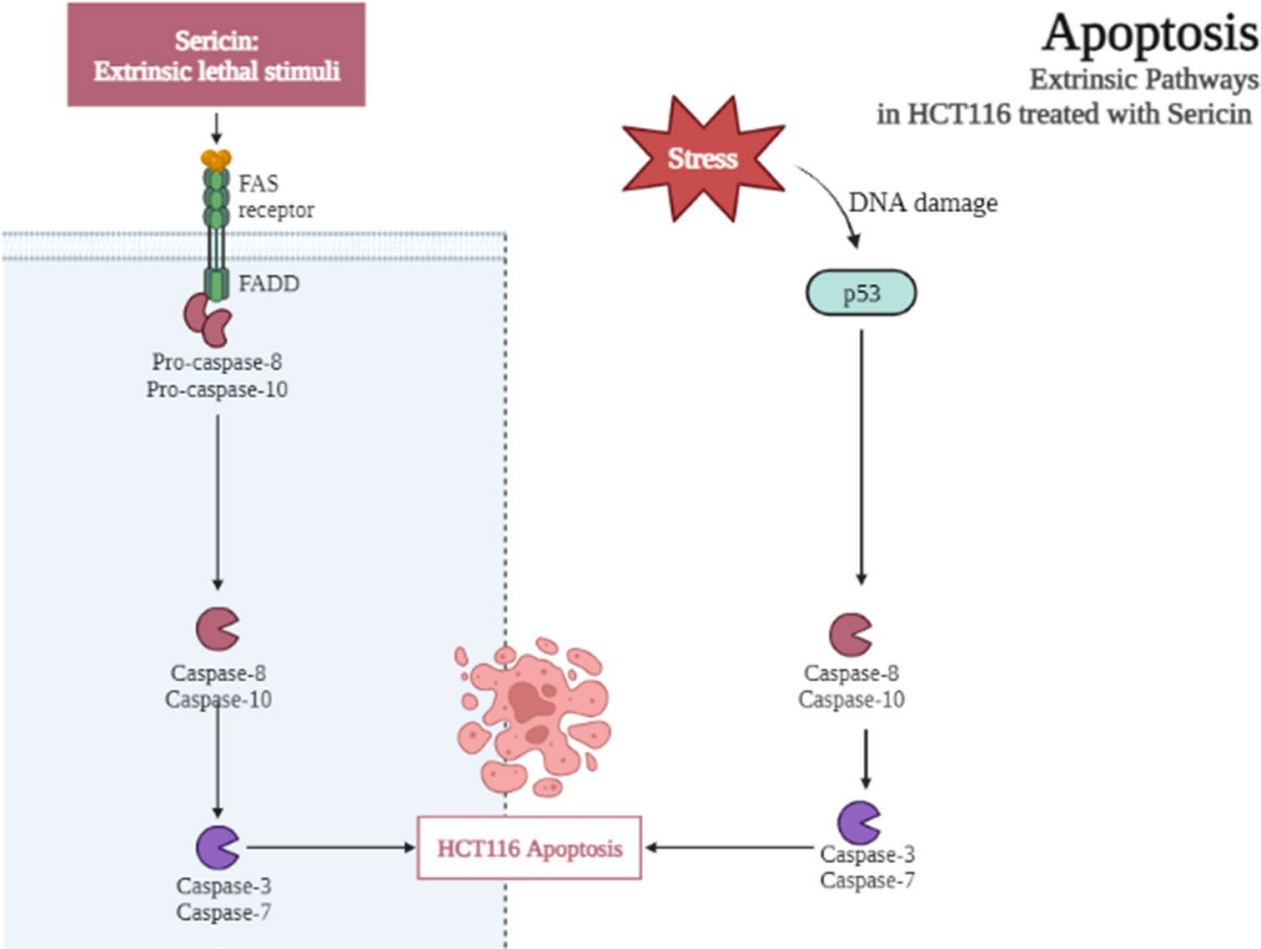
Diagram illustrating the extrinsic apoptosis pathway in HCT116 cells triggered by Sericin. Sericin instigated the extrinsic apoptosis pathway by engaging death receptors, leading to the activation of CASP8 and CASP10, subsequently triggering the functions of CASP3 and CASP7, ultimately inducing apoptosis in HCT116 cells. This picture was generated using BioRender.com.

Our study indicates that Sericin, a natural polymer sourced from silkworms, demonstrated dose-dependent cytotoxic effects on HCT116 cells, inducing apoptosis in HCT116 cells at higher concentrations. Furthermore, S-FTIR spectroscopy analysis revealed significant changes in macromolecules following Sericin treatment. The data showed a decrease in DNA content in Sericin-treated cells (Fig. 3e), which is associated with apoptosis in HCT116 cells induced by Sericin. Additionally, lipid content in Sericin-treated cells exhibited minimal changes (Fig. 3c). Transcriptomics analysis identified that Sericin induces apoptosis by activating the death receptor pathway, involving *TNFSF10* or *FASLG*, and triggering the expression of the *CASP8*, *CASP10*, *CASP3*, and *CASP7* genes. Early apoptotic protein analysis supported the data, indicating that Sericin activates p53, leading to an increase in CASP8, which, in turn, activates CASP3 and causes cell death in HCT116 cells. Moreover, the proteomics profiles revealed an upregulation of ELOVL5, suggesting a potential cause for the observed increment in lipid content. In summary, our findings suggest that Sericin influences apoptosis in HCT116 cells through the death receptor pathway, initiating the extrinsic apoptosis signaling pathway. This research holds promise for the development of innovative cancer treatment strategies incorporating Sericin. Figure 7 represents the schematic of the apoptosis extrinsic pathway in HCT116 cells triggered by Sericin.

## Material and methods

### Cell culture

This study used four cancer cell lines: non-small cell lung cancer NCI-H1975 (ATCC CRL-5908™), cholangiocarcinoma RBE (Cellosaurus (Expasy); CVCL_4896), hepatocellular carcinoma HepG2 (ATCC HB-8065™), and colorectal carcinoma HCT116 (ATCC CCL-247™). NCI-H1975 and RBE cells were cultured with RPMI medium (Gibco™), which contained 10% FBS (Gibco™) and 1% antibiotics (Gibco™). HepG2 and HCT116 were cultured with EMEM (ATCC; 30–2003™) or McCoy's 5A medium (Merck) supplemented with 10% FBS and 1% antibiotics. All cells were incubated in a 5% CO_2_ incubator and subcultured twice per week.

### Fifty percent inhibitory concentration (IC_50_) determination

The IC_50_ determination was used to clarify the cytotoxic effect of Sericin on cancer cells. Sericin was extracted by acid, alkaline, heat, and urea. The samples were used to determine the IC_50_ values against four cancer cell lines: NCI-H1975, RBE, HepG2, and HCT116. NCI-H1975 and RBE cells were seeded at a density of 5 × 10^4^ cells/mL in a 96-well plate. HepG2 and HCT116 cells were seeded at a density of 1 × 10^5^ cells/mL in a 96-well plate. All cells were incubated in a CO_2_ incubator overnight. Next, the sericin sample was diluted with culture medium to the desired concentration and treated with cancer cells for 72 h^59^. Afterward, the culture medium was removed and replaced with a culture medium containing PrestoBlue™ Cell Viability Reagent, Invitrogen™ (1:10 dilution). The cells were incubated in a CO_2_ incubator for 1 h and measured for absorbance by a microplate reader (BioTek Synergy™ HTX Multi-Mode Microplate Reader) at wavelengths of 570 nm. Therefore, the intensity of the colored product is directly correlated with the quantity of viable cells within the culture. The percent cell survival was determined using the subsequent Eq. (1) ^60^. The GraphPad Prism program (GraphPad Software Inc., San Diego, CA, USA) calculated the IC_50_ values.

1 $$\mathrm{\% Cell \, Survival }= \left(\frac{\mathrm{Number \, of \, viable \, cells \, after \ ,treatment}}{\mathrm{Number \, of \, viable \, cells \, in \, the control \, group}}\right)\times 100$$

### Apoptosis detection

The Muse™ Annexin V & Dead Cell Kit (MCH100105, EMD Millipore, Billerica, MA, USA) was used to detect HCT116 cell apoptosis by following the manufacturer’s instructions. HCT116 cells were seeded at a density of 2.5 × 10^5^ cells/mL in a 6-well plate overnight. The Sericin sample extracted by urea was diluted to 45 and 100 µg/mL in McCOY’s 5A complete medium. Next, remove the culture medium and replace it with a fresh medium containing sericin at the desired concentration. Doxorubicin at a concentration of 0.96 µM^46^ and untreated cells were used as positive and negative controls, respectively. HCT116 cells were incubated in a 5% CO_2_ incubator for 72 h. Subsequently, the treated cells were harvested by trypsinization and suspended in a fresh complete medium. Cells were stained with Muse® Annexin V and Dead Cell Kit (Luminex Corporation) and incubated at room temperature for 20 min in the dark. Fluorescence intensity was measured by flow cytometry using a Muse™ Cell Analyzer (Merck, Germany). The cells that were stained were put into four groups: live (annexin V−/7-AAD−), early apoptotic (annexin V+/7-AAD−), late apoptotic (annexin V+/7-AAD+), and necrotic (annexin V−/7-AAD+).

### Synchrotron Fourier transform Infrared (S-FTIR) spectroscopy

This technique is used to determine the macromolecule changes (lipid, protein, and nucleic acid) in the cells^61^. HCT116 cells were seeded at a density of 2.5 × 10^5^ cells/mL in a 6-well plate overnight. The Sericin sample was diluted to 26 and 34 µg/mL (based on IC_10_and IC_25_) in McCoy's 5A complete medium. The untreated cells were used as a control. The culture medium was removed and replaced with a 3 mL fresh medium containing Sericin. The treated cells were incubated in a 5% CO_2_ incubator for 72 h. Cells were harvested, trypsinized, and fixed with 10% formaldehyde for 30–60 min. After that, cells were washed with normal saline twice before being washed with distilled water three times. Cell pellets were suspended in 100–300 µL of distilled water, and 1–2 µL of cells were dropped onto BaF_2_ IR window 22 mm ϕ × 1 mm (Crystran, Poole, UK). The samples were dried in the desiccant cabinet for 2 h. Using S-FTIR microspectroscopy, it was possible to identify the change in biomolecules. S-FTIR experiments were completed at the Synchrotron Light Research Institute (a public organization), Nakhon Ratchasima, Thailand. The measurement system was prepared with a Vertex 70 FTIR spectrometer (Bruker Optics, Ettlingen, Germany) together with an IR microscope with 36 × objective lenses. To detect the biomolecule change, an MCT detector was used, and the detector was cooled with liquid nitrogen. The spectrometer used was the OPUS 7.8 from Bruker Optics in Ettlingen, Germany. It was set to transmission mode and had an aperture size of 10 × 10 μm^2^, a spectral resolution of 4 cm^−1^, and 64 scans over the measurement range of 4000 to 800 cm^−1^. Using OPUS 7.5, the sample group was taken from S-FTIR spectra with amide I absorption in the range of 0.2 to 1.2 arbitrary units. The Unscrambler × 10.5 (CAMO, Oslo, Norway) program performed principal component analysis (PCA) on the selected spectra.

### Nanostring-based transcriptomics

This experiment was used to determine the mRNA expression in HCT116-Sericin-treated cells. HCT116 cells were seeded in a 6-well plate at a density of 2.5 × 10^5^ cells/mL overnight. Cells were treated with 45 µg/mL Sericin for 24 h compared with untreated cells. Doxorubicin, at a concentration of 109 nM, was used as a positive control. Total RNA was extracted by TRIzol® Reagent (Invitrogen). Gene Expression CodeSet RNA Hybridization (Human PanCancer Pathways Panel; nanostring) was used to connect 200 µg of total RNA. The RNA was hybridized and incubated at 65 °C for 16 h. After that, 13 µL of sample were transferred to the nCounter SPRINT Cartridge and loaded into the nCounter SPRINT Profiler. The data were analyzed using NanoString nSolver 4.0 Ink Analysis Software. The internal standard genes (40 housekeeping genes, 8 negative controls, and 6 positive controls) were used to normalize the RNA count. The values of 0.958 and 0.961 are very close to 1, which represents a perfect prediction of the dependent variables (the experimental group) by the independent variables (the genes). These results can be linked to a qualified RNA preparation. The RNA preparation is a critical aspect of RNA sequencing studies, as it has a direct impact on the accuracy and consistency of the RNA count data. In the case where the correlation analysis reveals a high R^2^ value, such as > 0.95, it is indicative of a strong linear relationship between the RNA count data from the two groups.

### Early apoptotic protein detection

The MILLIPLEX® Early Apoptosis 7-Plex detection was conducted to confirm the effect of Sericin on the early apoptotic protein expression in HCT116 cells. This method is performed by following the manufacturer’s instructions. The proteins were extracted using a combination of chemical and mechanical lysis methods, followed by previously established protocols with minor modifications^62^. Cell lysis buffer (Cat: 9803; Cell Signaling Co., USA) was used to break up the HCT116 cells. It has 20 mM Tris–HCl (pH 7.5), 150 mM NaCl, 1 mM Na_2_EDTA, 1 mM EGTA, 1% TritonX-100, 2.5 mM sodium pyrophosphate, 1 mM β-glycerophosphate, 1 mM Na_3_VO_4_, and 1 µg/mL leupeptin. The filtered protein lysates were diluted 1:1 in MILLIPLEX® Assay Buffer. Fifty microliters of the assay buffer were added to each well and shaken at room temperature for 10 min. Decant assay buffer and added 25 µL of 1X beads to each well. After that, 25 µL of assay buffer (blank), control, and samples were added into the desired well. The reactions were incubated at 4 °C for 20 h with shaking. Afterward, the plate was washed with 100 µL assay buffer twice and added with 1X MILLIPLEX detection antibody. The reactions were incubated at room temperature for 1 h with shaking in the dark. The detection antibody was removed, and 25 µL of 1X Streptavidin-PE (SAPE) was added. The reaction was incubated at room temperature for 15 min with shaking in the dark. Twenty-five microliters of amplification buffer were added to the wells and incubated at room temperature for 15 min with shaking in the dark. Streptavidin-PE/Amplification Buffer was removed, and the beads were resuspended in 150 µL Assay Buffer. Read results using the appropriate Luminex® instrument.

### Proteomics analysis

Proteomics analysis is used to provide insights into the protein profiles of Sericin-treated cells. The proteins were extracted by using a combination of chemical and mechanical lysis methods, followed by previously established protocols with minor modifications^62^. The samples were sonicated using a tip-probe sonicator device for 3 cycles (3 s/3 s, 80% amplitude). The samples were centrifuged at 15,000×*g* for 10 min at room temperature. Only the supernatant was used for protein quantification using the BCA protein assay. A total of 200 µg of protein content from all samples was precipitated using an ice-cold 5% TCA/acetone solution (5% TCA, 0.07% β-mercaptoethanol, in acetone) at 1:10 (v/v) for 16 h at − 20 °C. The pallet protein was collected by centrifugation at 15,000×*g* for 10 min at room temperature and reconstituted in a solubilization solution (0.2% RapiGest SF, 5 mM NaCl, 10 mM NH_3_HCO_3_). A total of 25 µg of protein from all samples was eluted at 50 °C for 50 min using 5 mM TCEP. After that, samples were alkylated for 25 min at room temperature in the dark using 15 mM iodoacetamide. The samples were cleaned up using Thermo Scientific™ Zeba™ Spin Desalting Columns according to the manufacturer’s instructions. For protein digestion, a mixture of trypsin and Lys-C (1:50 w/w to sample protein content) was used. Peptide content was measured using Pierce™ Quantitative Peptide Assays (Cat: 23275, Thermo), and aliquots containing 20 µg of peptide per sample were dried and frozen at − 80 °C until LC–MS/MS analysis.

The data was acquired using an Orbitrap HF Mass Spectrometer (Thermo) coupled to an Easy nLC 1200 (Thermo). A 75 µm PepMap™ RSLC column with 2 µm C18 has a length of 50 cm. Binary LC was used; mobile A consisted of 0.1% formic acid, and mobile B consisted of 90% acetonitrile in 0.1% formic acid. The samples were separated using a 100 min linear gradient from 4 to 45% in mobile phase B, followed by a 15 min linear gradient from 45 to 90% in mobile phase B. We used both a data-independent (DIA) acquisition method and a higher-energy collisional dissociation (HCD) method with a collision energy of 27 to look at the peptides. Full-scan (MS) mass spectra were acquired from m/z 385 to 1015. For the DIA condition, an AGC target is set at 1 × 10^6^ ions and a resolution of 30 k. The isolation window was fixed at 16 m/z. Protein identification and quantification were done using Spectronaut® software (Biognosys AG Co., Switzerland). The protein sequence was retrieved from the UniProtKB database (organism: Homo sapiens, 10 March 2023). The retrieved database's FASTA protein sequences served as the basis for creating the spectral library. Protein abundance was normalized by using precursor intensity^63^. For further analysis and visualization, the resulting protein abundance values were log2-transformed.

### Statistical analysis

All experiments were carried out in at least three independent replicates (n = 3), and all data were expressed as the means ± standard deviation. The statistical significance was determined by Duncan’s multiple range test (*p*-values < 0.05). We used the ProteinPilotTM software to do a one-way analysis of variance (one-way ANOVA) at the protein level for pairwise comparisons during the proteomic analysis. We used two different types of multiple-testing corrections, the Bonferroni and the Benjamini–Hochberg FDR corrections. For Milliplex early apoptosis analysis, we performed a multiple t-test (*p*-values < 0.05).

### Supplementary Information

Supplementary Information.

## Data Availability

The datasets used or analyzed during the current study are available from the corresponding author upon reasonable request.
