# Supplementary material for: Exploring the apoptotic effects of sericin on HCT116 cells through comprehensive nanostring transcriptomics and proteomics analysis
Source: Sci Rep. 2024 Jan 29;14:2366. doi: 10.1038/s41598-024-52789-8 (PMC10825148; doi:10.1038/s41598-024-52789-8)
Supplement: Supplementary file 1 — Supplementary Information. [file 41598_2024_52789_MOESM1_ESM.pdf]

Nanostring-based transcriptomics data processing

Raw data was assessed using quality assurance (QA) metrics to measure data quality. All samples that meet QA metric tests were converted to log2 value to help distribute assumptions and normalized using average expression level of the housekeeping genes.

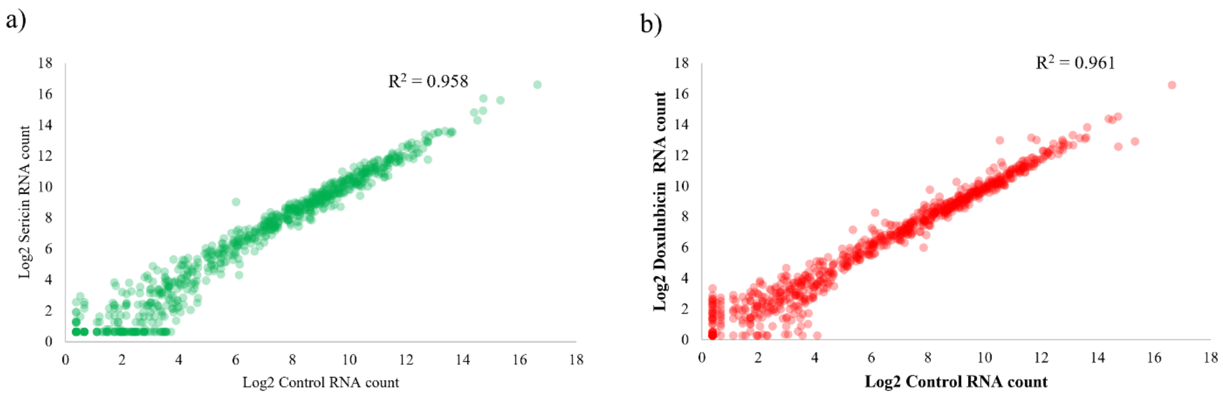

Figure S1: Scatter plot of log2 count RNA correlation analysis. a) Sericin versus control, b) Doxorubicin versus control.

Nanostring-based transcriptomics data pathway analysis

To explore the effect of Sericin and Doxorubicin in HCT116 cells, pathway analysis was conducted using Reactome analysis. Normalized counts from the Nanostring were uploaded onto ReactomeGSA (version 83) 3.7 using the "Pathway Analysis with Down-weighting of Overlapping Genes (PADOG)" algorithm to estimate the pathway and gene fold change score across all RNA sets analyzed [1]. The top 10 most differential pathways with p-value < 0.05 and FDR < 0.05 were reported. Unique and common significant pathways were illustrated in Venn-Diagram. The RNA expression analysis was showed in log2 fold change between Doxorubicin or Sericin and Control groups.

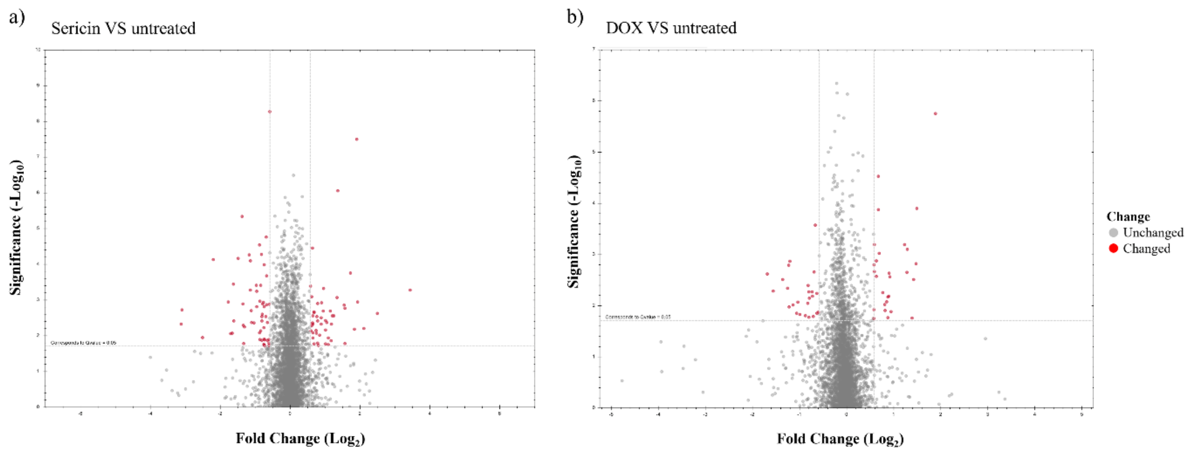

Figure S2: PADOG analyzed on HCT116 cells. Comparison of differential RNA abundance and pathway in Sericin and Doxorubicin versus Control groups. a) Differential RNA abundance in Sericin versus untreated, b) Differential RNA abundance in Doxorubicin versus untreated. DOX; Doxorubicin

Reference

1. Griss J, Viteri G, Sidiropoulos K, Nguyen V, Fabregat A, Hermjakob H: **ReactomeGSA - Efficient Multi-Omics Comparative Pathway Analysis**. *Mol Cell Proteomics* 2020, **19**(12):2115-2125.

The QC of proteomics dataset

The proteome dataset was used to identify and quantify proteins that differed according to sample conditions (with and without the Sericin). All samples were injection in 4-time and calculated the CV across these four replicates. Thresholds for a maximum CV typically range between 15 and 20% [2, 3]. In this experiment set a threshold CV at 20%. If the CV was higher than 20%, the data was excluded. The median CV across for untreatment was 13.7%, Doxorubicin 15.8, and Sericin 11.2% (range = 0–200%).

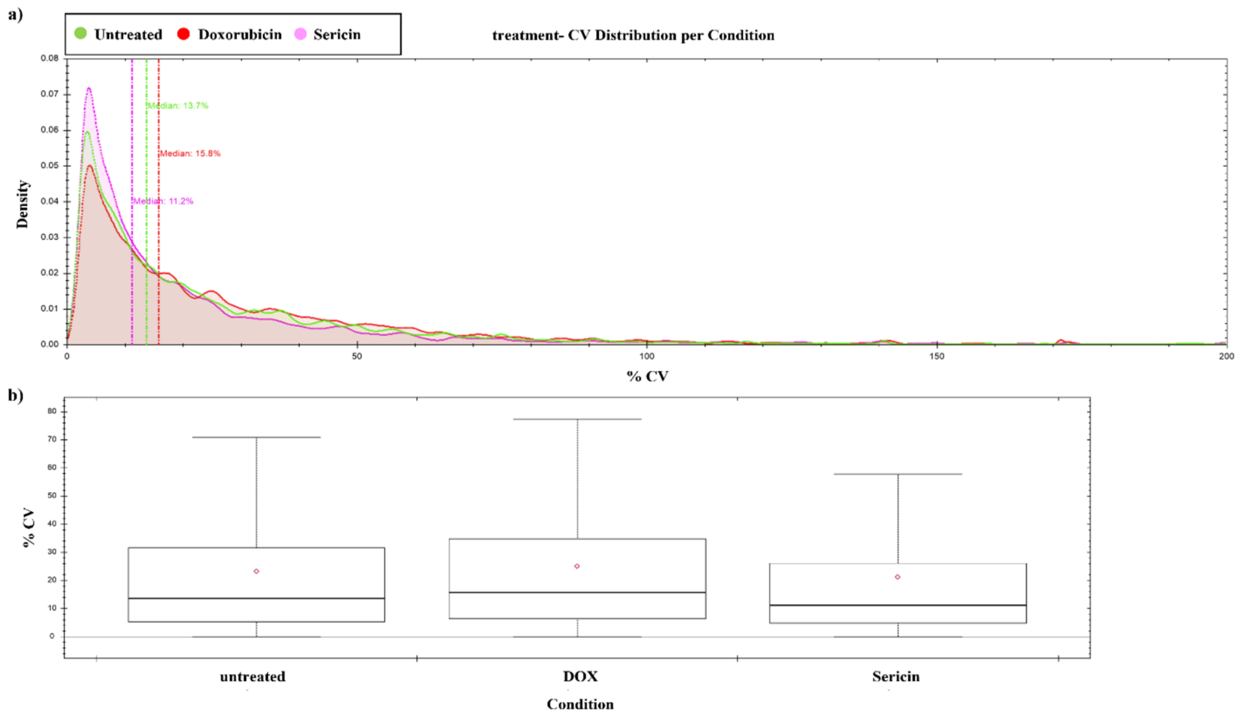

Figure S3: QC of the proteomics dataset. a) Coefficients of Variation shows the % CV distribution for all conditions in the experiment. b) Box-plot shows the distribution of CVs for each condition.

References

1. Liechti T, Kadelka C, Ebner H, Friedrich N, Kouyos RD, Gunthard HF, Trkola A: **Development of a high-throughput bead based assay system to measure HIV-1 specific immune signatures in clinical samples**. *J Immunol Methods* 2018, **454**:48-58.
2. Andreasson U, Perret-Liaudet A, van Waalwijk van Doorn LJ, Blennow K, Chiasserini D, Engelborghs S, Fladby T, Genc S, Kruse N, Kuiperij HB *et al*: **A Practical Guide to Immunoassay Method Validation**. *Frontiers in neurology* 2015, **6**:179.

Table S1 Transcriptomics expression data from nanostring

	expression data		
Gene Name	Doxorubicin	Sericin	untreated
AKT1	1.08	-0.18	-0.9
AKT2	1.15	-0.68	-0.47
AKT3	-0.02	1.01	-0.99
APC	-0.45	1.15	-0.7
BAD	-0.84	1.11	-0.26
BAX	1.05	-0.1	-0.94
BCL2	-0.7	1.14	-0.44
BCL2L1	-0.37	1.13	-0.76
BID	1.15	-0.61	-0.54
CASP10	-0.59	1.15	-0.57
CASP3	-0.74	1.14	-0.39
CASP7	0.87	0.23	-1.09
CASP8	0.98	0.03	-1.02
CASP9	1.08	-0.9	-0.18
EGFR	0.15	0.92	-1.07
ENDOG	-0.32	1.12	-0.8
FAS	1.15	-0.59	-0.57
FASLG	-0.78	1.13	-0.35
JAK1	-0.6	1.15	-0.56
JAK2	0.48	-1.15	0.67
JAK3	0.92	0.14	-1.06
JUN	-0.14	1.06	-0.92
MAP2K1	0.91	0.15	-1.07
MAP2K2	0.11	0.94	-1.05
MAP2K4	-0.9	1.08	-0.18
MAP3K1	1	0	-1
MAPK1	0.41	-1.14	0.73
MAPK3	0.83	0.28	-1.11
MAPK8	0.6	-1.15	0.55
MAPK9	0.28	-1.11	0.83
MTOR	-0.71	1.14	-0.43
MYD88	0.8	0.32	-1.12
PIK3CA	0.13	0.93	-1.06
PIK3CB	-0.82	1.11	-0.29
PIK3R1	0.92	-1.07	0.15
PIK3R2	0.97	0.06	-1.03
PTEN	1.15	-0.61	-0.54
SMAD2	0.95	-1.04	0.09
SMAD3	-0.28	1.11	-0.83
SOCS2	-1.15	0.49	0.66
SOCS3	-0.88	1.09	-0.21

STAT1	0.45	0.69	-1.15
STAT3	1.15	-0.6	-0.55
TGFBR2	1.12	-0.8	-0.32
TNFRSF10A	1.05	-0.94	-0.1
TNFSF10	-0.48	1.15	-0.67

Table S2 Proteomics results Sericin versus untreated

ProteinGroups	AVG Log2 Ratio	Pvalue	Qvalue	Genes	ProteinDescriptions
Q96RQ1	3.438526967	0.000538	0.007963	ERGIC2	Endoplasmic reticulum-Golgi intermediate compartment protein 2
Q96EP0	2.502983914	0.002429	0.016314	RNF31	E3 ubiquitin-protein ligase RNF31
Q8WXR4	2.112377712	0.00635	0.026802	MYO3B	Myosin-IIIb
Q10471	1.935015094	0.001133	0.011413	GALNT2	Polypeptide N-acetylgalactosaminyltransferase 2
Q9NYP7	1.908873781	3.19E-08	0.00011	ELOVL5	Elongation of very long chain fatty acids protein 5
A6NIH7	1.84841232	0.006617	0.027504	UNC119B	Protein unc-119 homolog B
Q96CU9	1.728815933	0.000177	0.004987	FOXRED1	FAD-dependent oxidoreductase domain-containing protein 1
Q9Y371	1.579148307	0.001707	0.013663	SH3GLB1	Endophilin-B1
Q9BRJ7	1.571798574	0.016781	0.04596	NUDT16L1	Tudor-interacting repair regulator protein
O15156	1.555732954	0.001401	0.012444	ZBTB7B	Zinc finger and BTB domain-containing protein 7B
P62891;Q59GN2	1.373296024	8.79E-07	0.000547	RPL39;RPL39P5	60S ribosomal protein L39;Putative 60S ribosomal protein L39-like 5
P53675	1.343457463	0.000857	0.009915	CLTCL1	Clathrin heavy chain 2
Q9NXK8	1.254550087	0.002822	0.017631	FBXL12	F-box/LRR-repeat protein 12
Q6NW29	1.206262879	0.002807	0.017631	RWDD4	RWD domain-containing protein 4
Q9NX08	1.185591733	0.004805	0.0233	COMMD8	COMM domain-containing protein 8
Q9Y620	1.176307026	0.013954	0.041489	RAD54B	DNA repair and recombination protein RAD54B
P08651	1.157299603	0.002036	0.015088	NFIC	Nuclear factor 1 C-type
Q6P161	1.101073028	0.018182	0.047956	MRPL54	39S ribosomal protein L54, mitochondrial
Q6P444	1.097507718	0.011606	0.037543	MTFR2	Mitochondrial fission regulator 2
O75600	1.054095166	0.004331	0.021876	GCAT	2-amino-3-ketobutyrate coenzyme A ligase, mitochondrial
Q9NVQ4	1.024702707	0.006931	0.028164	FAIM	Fas apoptotic inhibitory molecule 1
Q9Y2X9	1.02424075	0.003797	0.020482	ZNF281	Zinc finger protein 281
Q9UBV2	1.018763442	0.017136	0.046319	SEL1L	Protein sel-1 homolog 1
Q9Y618	0.957191017	0.000477	0.007468	NCOR2	Nuclear receptor corepressor 2
Q9ULD2	0.950013198	0.00207	0.015134	MTUS1	Microtubule-associated tumor suppressor 1
Q9NZM5	0.911296973	0.001216	0.01166	NOP53	Ribosome biogenesis protein NOP53
Q08AD1	0.897137541	0.004735	0.023115	CAMSAP2	Calmodulin-regulated spectrin-associated protein 2
Q9Y4X5	0.894142161	0.003143	0.018492	ARIH1	E3 ubiquitin-protein ligase ARIH1
P29372	0.843891893	0.009871	0.034219	MPG	DNA-3-methyladenine glycosylase

Q8N1G2	0.816474728	0.018848	0.048962	CMTR1	Cap-specific mRNA (nucleoside-2'-O-)-methyltransferase 1
O75695	0.796432855	0.016275	0.045212	RP2	Protein XRP2
Q8N0U8	0.794088198	0.003879	0.020673	VKORC1L1	Vitamin K epoxide reductase complex subunit 1-like protein 1
Q96S52	0.788496107	0.001272	0.011868	PIGS	GPI transamidase component PIG-S
Q9UN36	0.765482399	0.002908	0.017937	NDRG2	Protein NDRG2
Q9UL45	0.75691693	0.008573	0.032036	BLOC1S6	Biogenesis of lysosome-related organelles complex 1 subunit 6
O00399	0.752583848	0.007196	0.02895	DCTN6	Dynactin subunit 6
P21589	0.69064709	0.002178	0.015523	NT5E	5'-nucleotidase
O60942	0.6873106	0.002289	0.015851	RNGTT	mRNA-capping enzyme
Q8NEG4	0.687203171	0.017141	0.046319	FAM83F	Protein FAM83F
O43324	0.661905362	0.002821	0.017631	EEF1E1	Eukaryotic translation elongation factor 1 epsilon-1
Q9NUL7	0.658309264	0.011736	0.037764	DDX28	Probable ATP-dependent RNA helicase DDX28
Q02750	0.656928646	0.008479	0.031845	MAP2K1	Dual specificity mitogen-activated protein kinase kinase 1
P57105	0.651375269	0.004773	0.023221	SYNJ2BP	Synaptojanin-2-binding protein
P82912	0.640986088	0.00439	0.022059	MRPS11	28S ribosomal protein S11, mitochondrial
P21964	0.640806182	3.53E-05	0.002541	COMT	Catechol O-methyltransferase
Q9NX04	0.623285982	0.000821	0.009735	C1orf109	Ribosome biogenesis protein C1orf109
Q92974	0.619799162	0.005325	0.024447	ARHGEF2	Rho guanine nucleotide exchange factor 2
Q8NCF5	0.596868918	0.007376	0.029454	NFATC2IP	NFATC2-interacting protein
Q8N999	0.585615777	0.000411	0.006998	C12orf29	Uncharacterized protein C12orf29
P35527	-0.582317042	5.41E-09	3.73E-05	KRT9	Keratin, type I cytoskeletal 9
P09234	-0.584658509	0.001326	0.012138	SNRPC	U1 small nuclear ribonucleoprotein C
O75352	-0.595230105	0.013065	0.040044	MPDU1	Mannose-P-dolichol utilization defect 1 protein
Q9UJK0	-0.600905981	0.016447	0.045525	TSR3	18S rRNA aminocarboxypropyltransferase
Q9HBU6	-0.601919384	0.018745	0.048841	ETNK1	Ethanolamine kinase 1
O14880	-0.640979081	0.013721	0.041211	MGST3	Microsomal glutathione S-transferase 3
Q13188	-0.649093085	0.004294	0.021817	STK3	Serine/threonine-protein kinase 3
P03915	-0.670876256	0.000212	0.005345	MT-ND5	NADH-ubiquinone oxidoreductase chain 5
Q9Y3E0	-0.671783367	0.001381	0.012412	GOLT1B	Vesicle transport protein GOT1B
P52435;Q9GZM3;Q9H1A7	-0.681899776	1.76E-05	0.00181	POLR2J;POLR2J2;POLR2J3	DNA-directed RNA polymerase II subunit RPB11-a;DNA-directed RNA polymerase II subunit RPB11-b1;DNA-directed RNA polymerase II subunit RPB11-b2

Q16775	-0.690830128	0.002967	0.018177	HAGH	Hydroxyacylglutathione hydrolase, mitochondrial
Q01167	-0.725792181	0.019234	0.049395	FOXK2	Forkhead box protein K2
Q9NQ55	-0.728596948	0.013146	0.040256	PPAN	Suppressor of SWI4 1 homolog
Q9Y6I9	-0.730082264	0.018288	0.048087	TEX264	Testis-expressed protein 264
Q86UV5	-0.730426227	0.000105	0.004095	USP48	Ubiquitin carboxyl-terminal hydrolase 48
Q86UY8	-0.734920438	0.001574	0.013153	NT5DC3	5'-nucleotidase domain-containing protein 3
Q9P2X0	-0.735000379	0.012726	0.039409	DPM3	Dolichol-phosphate mannosyltransferase subunit 3
Q9Y3D5	-0.750210099	0.017645	0.046973	MRPS18C	28S ribosomal protein S18c, mitochondrial
P11509;Q16696	-0.750840939	0.006056	0.025991	CYP2A6;CYP2A13	Cytochrome P450 2A6;Cytochrome P450 2A13
Q53ET0	-0.761355033	0.00118	0.011576	CRTC2	CREB-regulated transcription coactivator 2
P02533	-0.777945327	0.001303	0.012007	KRT14	Keratin, type I cytoskeletal 14
Q9BYC9	-0.78784535	0.002507	0.016613	MRPL20	39S ribosomal protein L20, mitochondrial
Q8N5D0	-0.797965631	0.002922	0.017973	WDTC1	WD and tetratricopeptide repeats protein 1
O14735	-0.800115865	0.013474	0.040885	CDIPT	CDP-diacylglycerol--inositol 3-phosphatidyltransferase
Q99941	-0.805548504	0.003896	0.020707	ATF6B	Cyclic AMP-dependent transcription factor ATF-6 beta
Q53HC9	-0.816778482	5.39E-05	0.002945	EIPR1	EARP and GARP complex-interacting protein 1
Q6YP21	-0.839985406	0.007446	0.02956	KYAT3	Kynurenine--oxoglutarate transaminase 3
P62273	-0.841082874	0.00039	0.006892	RPS29	40S ribosomal protein S29
P00747	-0.855551844	0.012905	0.039714	PLG	Plasminogen
Q06033	-0.865419745	0.001101	0.011349	ITIH3	Inter-alpha-trypsin inhibitor heavy chain H3
P06241	-0.866352436	0.008347	0.031675	FYN	Tyrosine-protein kinase Fyn
P53634	-0.868008846	2.93E-05	0.002347	CTSC	Dipeptidyl peptidase 1
P01116	-0.94587015	0.001542	0.013057	KRAS	GTPase KRas
O15533	-0.949880789	0.000573	0.008189	TAPBP	Tapasin
Q9BTY2	-0.959519467	0.00039	0.006892	FUCA2	Plasma alpha-L-fucosidase
Q7Z309	-1.026330829	0.004403	0.022091	PABIR2	PABIR family member 2
O75323	-1.099970514	0.004327	0.021872	NIPSNAP2	Protein NipSnap homolog 2
P13647	-1.114183813	0.001985	0.01499	KRT5	Keratin, type II cytoskeletal 5
Q8N5G0	-1.127681486	8.09E-05	0.003461	SMIM20	Small integral membrane protein 20
P13929	-1.128878903	0.000528	0.007878	ENO3	Beta-enolase
Q13362	-1.163162577	5.57E-05	0.002997	PPP2R5C	Serine/threonine-protein phosphatase 2A 56 kDa regulatory subunit gamma isoform

Q99988	-1.297630945	0.005747	0.025299	GDF15	Growth/differentiation factor 15
Q9BX95	-1.326880616	0.01677	0.045958	SGPP1	Sphingosine-1-phosphate phosphatase 1
Q9BVA0	-1.330604508	0.00514	0.024136	KATNB1	Katanin p80 WD40 repeat-containing subunit B1
Q8WV99	-1.341805432	0.001301	0.012007	ZFAND2B	AN1-type zinc finger protein 2B
P55199	-1.374925456	4.58E-06	0.001011	ELL	RNA polymerase II elongation factor ELL
P02649	-1.48642909	6.84E-05	0.003248	APOE	Apolipoprotein E
O95298	-1.606247394	0.003901	0.020707	NDUFC2	NADH dehydrogenase [ubiquinone] 1 subunit C2
P21741	-1.621608415	0.000366	0.006763	MDK	Midkine
Q9H9E3	-1.651441594	0.008575	0.032036	COG4	Conserved oligomeric Golgi complex subunit 4
Q9BZV1	-1.698764839	0.008756	0.032405	UBXN6	UBX domain-containing protein 6
P27105	-1.762287423	0.001137	0.011413	STOM	Stomatin
P34059	-2.194797529	7.40E-05	0.003315	GALNS	N-acetylgalactosamine-6-sulfatase
Q13586	-2.505605217	0.011389	0.037246	STIM1	Stromal interaction molecule 1
A8MWD9;P62308	-3.089198896	0.00189	0.014573	SNRPGP15;SNRPG	Putative small nuclear ribonucleoprotein G-like protein 15;Small nuclear ribonucleoprotein G
P47914	-3.116053219	0.004811	0.0233	RPL29	60S ribosomal protein L29
